# Spin Vortex Resonance in Non-planar Ferromagnetic Dots

**DOI:** 10.1038/srep25196

**Published:** 2016-05-04

**Authors:** Junjia Ding, Pavel Lapa, Shikha Jain, Trupti Khaire, Sergi Lendinez, Wei Zhang, Matthias B. Jungfleisch, Christian M. Posada, Volodymyr G. Yefremenko, John E. Pearson, Axel Hoffmann, Valentine Novosad

**Affiliations:** 1 Materials Science Division, Argonne National Laboratory, Argonne, IL 60439, USA; 2 Texas A&M University, College Station, TX 77843, USA.

## Abstract

In planar structures, the vortex resonance frequency changes little as a function of an in-plane magnetic field as long as the vortex state persists. Altering the topography of the element leads to a vastly different dynamic response that arises due to the local vortex core confinement effect. In this work, we studied the magnetic excitations in non-planar ferromagnetic dots using a broadband microwave spectroscopy technique. Two distinct regimes of vortex gyration were detected depending on the vortex core position. The experimental results are in qualitative agreement with micromagnetic simulations.

The investigation of spin dynamics in geometrically confined ferromagnets is an important research topic due to both the technological and the basic science relevance. Some prominent examples include nanoscale spin textures with non-collinear magnetization arrangement such as skyrmions^1^, domain walls^2^,^3^, and spin vortices^4^. A vortex-type magnetization is energetically favorable for micron and sub-micron disks with no crystalline anisotropy. The long-range dipolar forces govern the spin dynamics in the vortex-state. Of particular interest is a low-frequency excitation mode associated with the vortex core gyration. Besides the magnetization of saturation, the vortex eigenfrequencies depend on the dot geometric aspect ratio^5^. In magnetic particles with an in-plane shape anisotropy, such as ellipses^6^, squares^7^ or triangles^8^ the resonance frequencies vary with not only the dot sizes, but also with the value of applied magnetic field. This is in sharp contrast to disk-shaped elements where the excitation spectrum depends rather weakly (<10%) on the value of an in-plane applied field^6^.

Furthermore, it has been reported that the intrinsic pinning due to grain boundaries^9^, surface roughness^10^, exchange bias^11^ or layered dot system^12^,^13^,^14^ could also influence the gyration of the vortex core. While the vast majority of the above mentioned works deal with geometrically flat elements, here we have studied circular elements with intentionally altered topography. This was achieved by using a pre-patterned substrate with the characteristic lateral dimensions significantly smaller than the diameter of the desired magnetic element. In our case, the nonmagnetic disks were first defined via a lift-off process, and then covered by another patterned ferromagnetic layer with the same geometric center but a larger diameter. As result, we obtained a “hat-like” sample structure,schematically depicted in Fig. 1(a). Then the dynamic properties of these non-planar elements were systematically investigated using broadband microwave spectroscopy and micromagnetic modeling as a function of their dimensions and amplitude of the external field. As we show below, the introduced disk-shaped step (also referred as a vortex barrier) provides a strong geometric confinement and vortex core pinning effect.

## Results

### Element geometry

**Figure 1:**
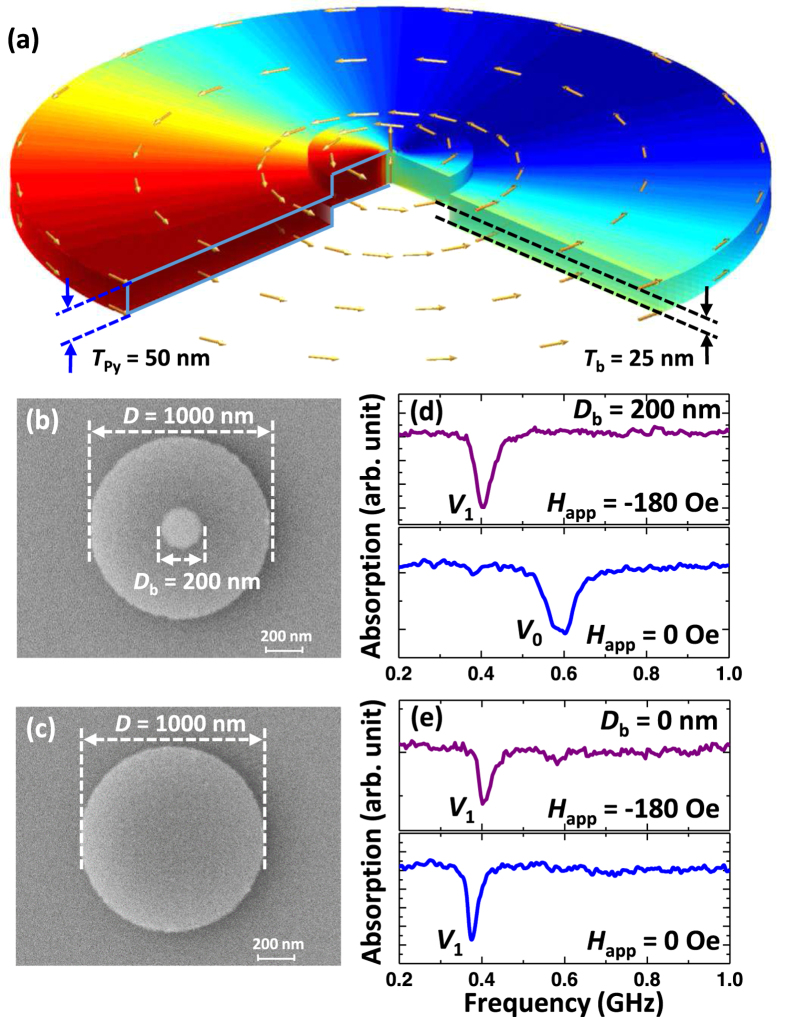
(**a**) A sketch of the dot with *D*_b_ = 200 nm with the simulated remnant magnetization state of the engineered dot. The x-component of the magnetization is represented from red to blue in a range of +1 to −1. Scanning electron micrographs of the dot with (**b**) 200 nm barrier diameter (*D*_b_) and (**c**) reference dot. (**d**,**e**) Shows the experimental FMR curves with *H*_app_ = −180 Oe and 0 Oe for *D*_b_ = 200 nm and 0 nm, respectively.

Two sets of samples were fabricated. The first is a reference sample, a flat Permalloy (Py, Ni_80_Fe_20_ alloy) disk with diameter of 1 micron, and thickness of 50 nm. The second is a non-planar, or “engineered” dot, which is also circular in shape, with the same dimensions but has altered topography. Shown in Fig. 1(a) is a sketch of the cross-section profile of the engineered dot in which the magnetic material at the center has been lifted 25 nm by preparing a non-magnetic (Titanium) island prior to the deposition of the ferromagnetic material. Figure 1(b,c) show representative scanning electron microscope images of the non-planar dot with 200 nm diameter of the step and the reference dot. In all samples presented in this paper, the step height *T*_b_ and the dot thickness *T*_Py_ have been fixed at 25 nm and 50 nm, respectively while the inner diameter *D*_b_ varied from 150 to 300 nm.

### Dynamic response

In order to characterize the dynamic response of the samples, a microwave transmission measurement has been performed on an array of equivalent dots in a broad frequency range using a vector network analyzer (VNA). Applying an external magnetic field *H*_app_ allowed us to probe how the dynamic response of the vortex core changes once it has been displaced from the central area of the disk.

Figure 1(d) compares the absorption spectra of the engineered dot taken at remanence (*H*_app_ = 0), and in applied field (*H*_app_ = −180 Oe) after saturating the sample with a –1500 Oe external field. Interestingly, for a higher value of applied field we detect the resonance frequency of the vortex translation mode (*V*_1_) at ~0.4 GHz, while for the *H*_app_ = 0 Oe the frequency (*V*_0_) increases almost 50%, to ~0.6 GHz. The same microwave measurements performed for a flat Ni_80_Fe_20_ dot (e.g. the reference sample, without a vortex barrier) are shown in Fig. 1(e). The frequency change when *H*_app_ is decreased from −180 Oe to 0 Oe is insignificant for the reference dot. A slightly higher frequency for a shifted vortex state is in fact expected. It originates from additional dipolar and exchange forces due the vortex spin structure deformation. The striking result is that while the resonance frequencies for *the shifted vortex* states are the same for both samples, their values *in remanence* differ significantly. This suggests that the dynamic response of the engineered dot can be fine-tuned by controlling the relative position of the vortex core with respect to the vortex barrier using a relatively small magnetic field. One could speculate that for the engineered dot subjected to *H*_app_ = −180 Oe, the vortex core is located outside the circumference of the barrier and thus it encounters the dipolar fields averaged within the outer boundary of the entire dot. As the |*H*_app_| value is reduced to zero, the vortex core shifts to the dot center and its gyration is now governed by the dominant contribution from the magnetic charges confined within the barrier boundary. As a result, there appears a ~0.2 GHz frequency difference between these two core positions. Obviously, this effect is not observed for the reference dot (Fig. 1(d)) due to the lack of the barrier.

**Figure 2:**
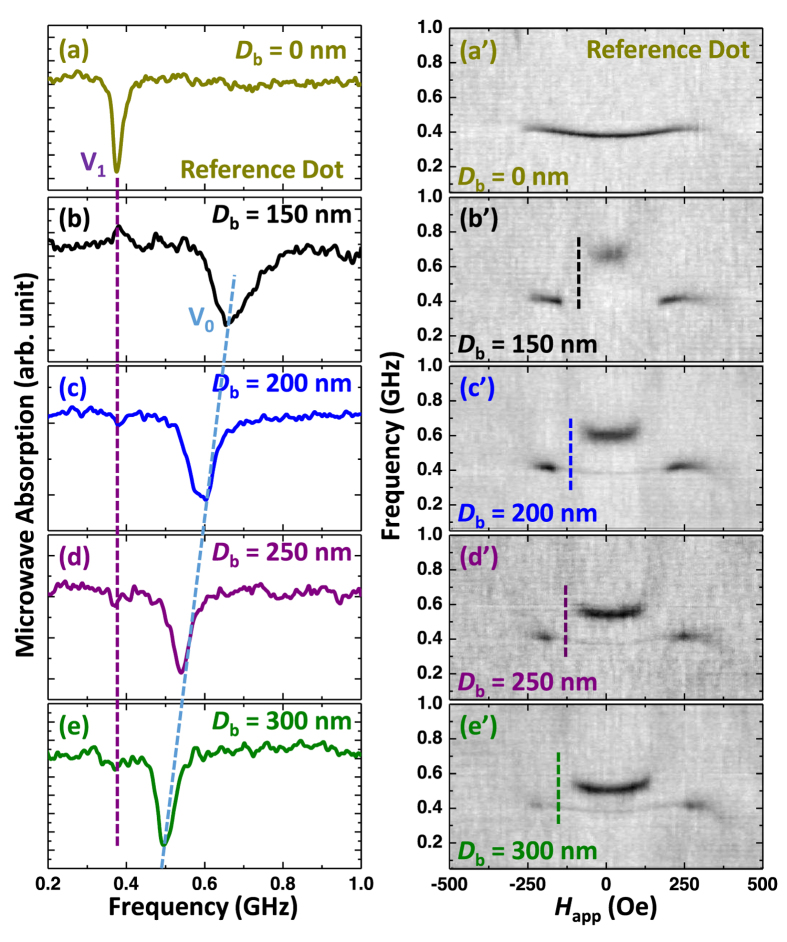
(**a**–**e**) The experimental remanent FMR absorption curves of the dots with *D*_b_ = 0 nm (reference dot), 150 nm, 200 nm, 250 nm, and 300 nm. The corresponding 2D FMR absorptions spectrums are shown in (**a’**–**e’**). The vertical lines in (**b’**–**e’**) indicate the field range where the vortex core is pinned at the barrier.

To further clarify the observed effect, we fabricated and systematically investigated the dots with variable barrier diameters. Figure 2(b–e) are the representative absorption spectra taken at remanence (*H*_app_ = 0 Oe) for the 50 nm thick, 1 micron diameter Py dots but with *D*_b_ varied from 150 to 300 nm. The vortex mode of all modified dots is characterized by the domination of one peak. The frequency of this main mode (*V*_0_) decreases as the barrier diameter is increased. This trend is similar to the results of previous studies^15^ for planar Ni_80_Fe_20_ disks where the resonance frequency was reported to decrease with increasing dot diameter (for a fixed thickness of the element). The micromagnetic modeling yields similar results as it is discussed below. It should be noticed, that the resonance linewidth is increased for smaller *D*_*b*_ values, consistent with the dependence of the vortex dynamic susceptibility and effective damping on the dot geometrical parameters^16^. Furthermore, additional resonance line broadening may originate from the fact that e-beam patterning precision inevitably worsens with the decrease of the barriers diameter. This causes gyrotropic frequency variation across the dot array, and thus the resonance broadening as seen in Fig. 2(b–e).

The 2D absorption spectra (Fig. 2(a’–e’)) also contain important information about the magnetization evolution process. For clarity, only the forward half of the results (with *H*_*app*_ increasing from negative to positive saturation field) is shown. The spectra asymmetry on the field sweep direction is related to the vortex nucleation and annihilation effects. Let us consider the reference dot (*D*_b_ = 0 nm, Fig. 2(a’)) and the dot with *D*_b_ = 200 nm (Fig. 2(c’)) as two examples. The vortex resonance corresponding to the translational mode appears at *H*_app_ ~250 Oe in both structures, indicating that the value of the vortex nucleation field is not affected by presence of the vortex barrier. Unlike the almost field-independent spectra of the reference dot, a discontinuous step-like frequency change is observed in the engineered dot. For instance, for *H*_app_ = −180 Oe and 0 Oe, the frequency difference is ~200 MHz for the dot with *D*_b_ = 200 nm (Fig. 1(d)), while it is only ~15 MHz for the reference dot (Fig. 1(e)). As mentioned before, this jump in frequency is attributed to changes in the position of the vortex core. In a high magnetic field, when *H*_app_ is just below the nucleation field, the vortex core is located close to the outer edge. As the magnetic field *H*_app_ is gradually decreased, the vortex core progressively displaces towards to the center of the dot. The intensity of the resonance line in an engineered dot remains unchanged till *H*_app_ = −1500 Oe. In the field range from −150 Oe to −60 Oe the signal disappears (or is below our experimental sensitivity limit). We speculate that in this fields range the vortex gyration is significantly suppressed due to the pinning effect of the barrier edges. (This vortex core pinning field range varies as a function of the barrier diameter as the vertical line indicates in Fig. 2(b’–e’)). The resonance reappears again when *H*_app_ is decreased below −60 Oe suggesting that the vortex core is now fully inside the barrier circumference. Stronger dynamic dipolar fields of the barrier cause the gyrotropic mode frequency to shift to a much higher values. The frequency does not change in small positive fields till the gyration stops when the core reaches the barrier edge again. With greater magnetic field, the vortex core overcomes the barrier border and the low frequency resonance line re-emerges again. Similarly to the nucleation fields, the vortex annihilations fields almost do not differ in the modified and reference dots. Thus, while we have seen that altering the dot topography has a profound impact on the low-field vortex core dynamics, its overall magnetostatic properties (e.g. the hysteresis loop) remain unaffected. This is different from the case of a dot-on-dot structure where bi-stable magnetic states were reported^12^.

### Micromagnetic modeling

**Figure 3:**
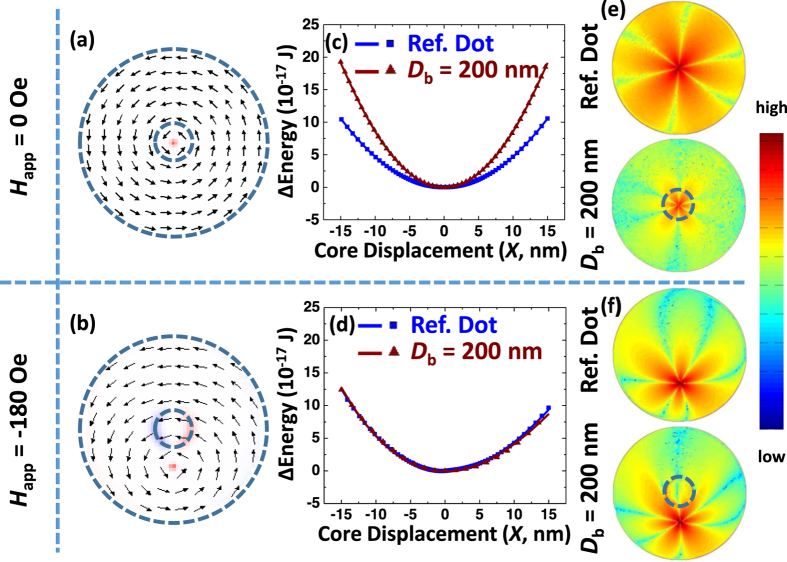
The simulated magnetization state (central layer) of the dot with *D*_b_ = 200 nm for *H*_app_ = 0 Oe (**a**) and −180 Oe (**b**). (**c**,**d**) Shows the plots of the simulated energy profile of both the reference dot and the engineered dot as a function of the core displacement for *H*_app_ = 0 Oe and −180 Oe. Solid lines are the fitting results of the profile with the parabolic function. The simulated magnetic charge density 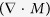 changes when the core is displaced from the equilibrium position (when it is performing gyrotropic movement) for the reference dot and the engineered dot with *H*_app_ = 0 Oe (**e**) and −180 Oe (**f**). The color code is in log scale.

A systematic micromagnetic study has been performed to further investigate the static and dynamic response of the modified dots. The simulations confirm that in spite of such significant alteration to the topography of the disk, the vortex state remains the ground state for the system. Figure 3(a,b) show the magnetization distribution in remanence for the dot with *D*_b_ = 200 nm and in an in-plane magnetic field *H*_app_ = −180 Oe.

A significant difference in the eigenfrequencies for these two distinct cases (e.g. the core located outside and inside of the barrier circumference) were also confirmed micromagnetically. Shown in Fig. 3(c) are the calculated relative energy profiles of the engineered dot (triangle symbols) and reference dot (square symbols) plotted as a function of the displacement of the vortex core. A clear difference between the two sets of results indicates the strong effect of the vortex confinement when the core is at the center of the element. The energy profile *vs* the core displacement can be approximated as a parabolic function^17^ *E*(*X*) = *E*(0) +1/2*κX*^2^, where *κ* is the effective stiffness coefficient, *E*(0) is the energy at the equilibrium position and *X* is the vortex core displacement. It should be noted that the magnetostatic energy provides the dominant contribution to *E*(*X*). Using the simulation data shown in Fig. 3(c), one can find the remanent values of stiffness coefficients (*κ*) as 0.94 × 10^−20 ^J/nm^2^ and 1.7 × 10^−20 ^J/nm^2^ for the reference and engineered dots, respectively. Since the frequency is directly proportional to *κ*^18^, the vortex core gyrates faster when trapped inside the barrier. There appears no significant difference between the energy profiles for engineered (triangle symbols) and reference (square symbols) dots when the vortex core is located outside the barrier (*H*_app_ = −180 Oe), Fig. 3(d). The asymmetry in the energy profile due to vortex structure deformation in the shifted state can be accounted by adding a cubic term^6^.

As the vortex core is very small, the dipolar forces originating from the dynamic magnetic charges outside the vortex core govern its gyration. These charges can be calculated using a so-called “side-surface charges free” analytical model^19^. Within this model, the magnetization distribution of a precessing vortex obeys boundary conditions such that there is no net magnetization component perpendicular to the dot’s lateral surface. Altering the topography that leads to formation of a step-like barrier will inevitably impose an additional requirement so the magnetic “charges” on the barrier edge surfaces are minimized as well. Figure 3(e) shows representative images of temporal changes in the divergence of the magnetization for flat and engineered dots (upper and lower images, respectively). It is clear that the volume magnetostatic charges contributing to the vortex dynamics and defining its eigenfrequency are well-distributed across the dot for the reference sample, while their counterparts in the engineered dot are predominantly located in the central area circumscribed by the barrier edge. The same simulation was performed for *H*_app_ = −180 Oe as shown in Fig. 3(f). To our surprise, in this case the spatial distribution of changes in the volume charges is almost identical for both samples resulting in similar vortex gyration frequencies.

**Figure 4:**
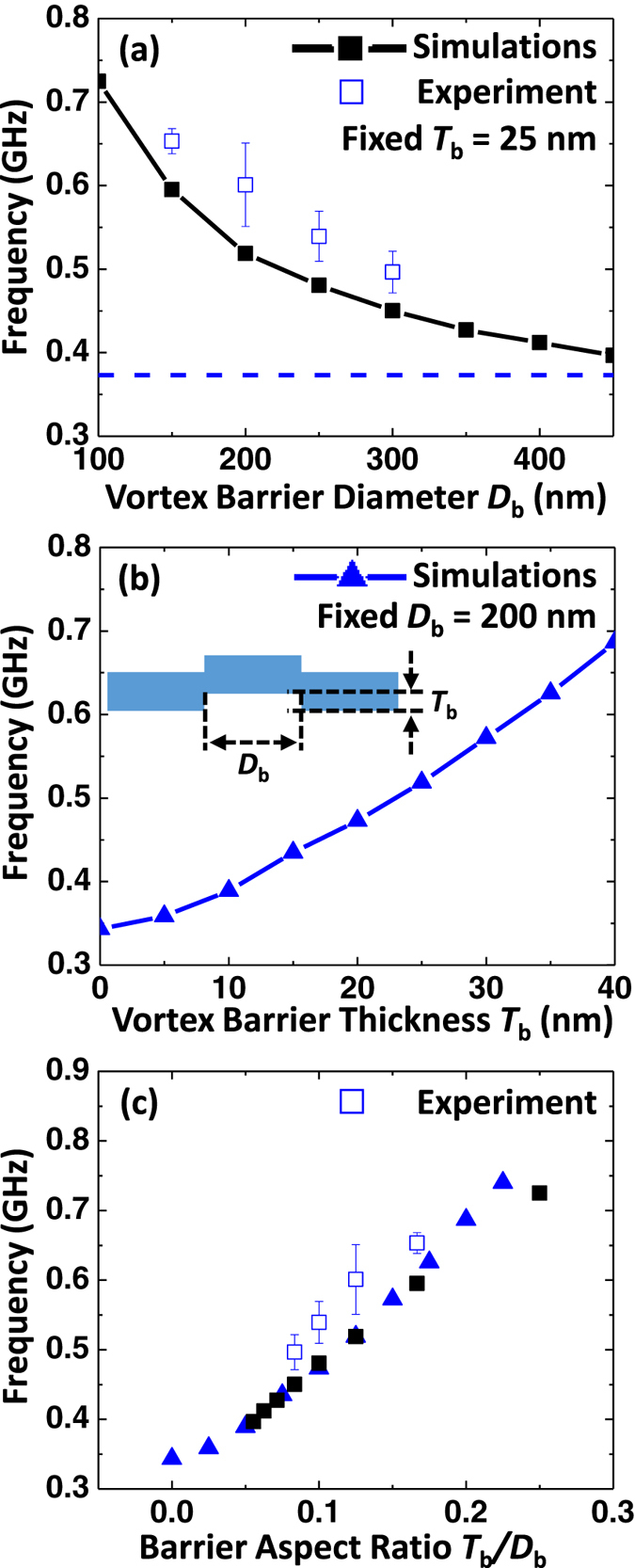
(**a**,**b**) Shows the simulated and experimental results of the gyrotropic mode frequency of the modified dots as a function of the barrier diameter (*D*_b_) and the barrier thickness (*T*_b_), respectively. (**c**) Shows all results re-plotted as a function of the barrier aspect ratio *T*_b_/*D*_b_.

Finally, to further understand the effect of the vortex barrier to the translational mode frequency, systematic simulations have been performed as a function of the barrier size. Shown in Fig. 4(a) is the summary of the experimental (square symbols) and micromagnetic (solid line) frequencies as a function of *D*_b_. While there is a noticeable quantitative discrepancy between the experimental and computational results, they are in good qualitative agreement. One explanation for this discrepancy is a possible difference between the barrier edge geometry (shape, and thickness) of the experimental sample, and its numerical model. The exact configuration of this shell may influence the coupling strength between the inner and outer parts of the dot, and thus influence the resulting resonant frequency. Figure 4(b) shows the simulated frequency plotted as a function of barrier thickness *T*_b_. The frequency continuously increases with *T*_b_, it almost doubles in comparison to the reference dot for *T*_b_ = 40 nm. These results demonstrate that the vortex resonance frequency can be effectively controlled by adjusting the barrier geometry. The low field experimental and micromagnetic frequencies for engineered dots shown in Fig. 3(a,b) scale universally when replotted as a function the barrier geometric aspect ratio *T*_b_*/D*_b_. Interestingly, this trend is similar to how the translational mode frequency in flat disks scales as a function of the disk thickness to diameter ratio^15^.

## Summary

A nonmagnetic nanodot inserted under a mesoscale Ni_80_Fe_20_ dot was shown to provide a geometric confinement effect causing changes in the vortex translational mode frequency. Two distinct resonance frequency ranges were observed depending on the position of the vortex core (inside or outside of the barrier) controllable by applying a relatively small magnetic field. By comparing the experimental data and micromagnetic simulations, it was found that the frequency of the gyrotropic mode increases as the thickness-diameter ratio of the barrier is increased. Further studies of such non-planar ferromagnetic elements will be focused on the details of the pinning mechanism, its possible impact on the energetics of the vortex core reversal process, and high frequency spin dynamics.

## Methods

### Sample fabrication

The engineered dots were fabricated using a multistep electron-beam (EBL) lithography process. First, the disk arrays with diameter in a range of 150 nm to 300 nm and alignment marks were defined on polymethyl methacrylate (PMMA) resist, accompanied by e-beam evaporation and lift-off process of a 25-nm-thick titanium film. The second step EBL patterning of 1-μm diameter disks followed by deposition of 50-nm-thick Ni_80_Fe_20_ and lift-off completes the fabrication process. The barriers and the disks are concentric as is confirmed by Scanning Electron Microscopy (SEM) imaging. For each type of sample, the collective response of ~500 dots has been measured. The inter-dot distance is fixed at 2-μm for all arrays. For such a large edge-to-edge distance, the dipolar interaction between the neighboring elements is negligible and thus can be ignored^20^.

### Spectral measurements

In order to characterize the dynamic properties of the samples, a coplanar waveguide (CPW) with a 3 μm-wide-signal line was fabricated on top of each dot array using optical lithography followed by Ti(5 nm)/Au(150 nm) sputter deposition and a lift-off process. Microwave transmission measurements have been performed in the 0.05 ~ 10 GHz frequency range using a broadband microwave vector network analyzer (VNA). The microwave transmission was measured by sweeping the frequency in a fixed magnetic field. Applying an in plane magnetic field allowed us to probe how the dynamic response of the vortex core changes once it has been displaced from the central area of the disks. Since the focus of this paper is the fundamental gyrotropic vortex mode, all results are presented in the field range of −500 ~ 500 Oe and frequency range of 0.05 ~ 1.0 GHz. Prior to the magnetic field sweep, the samples were magnetized at 1500 Oe field.

### Micromagnetic simulation details

Systematic micromagnetic modeling was performed using mumax3 code^21^. Typical parameters for Ni_80_Fe_20_ (the saturation magnetization *M*_s_ = 700 emu/cm^3^, the exchange constant *A* = 130 μerg/cm, the damping factor 0.01, the gyromagnetic ratio *γ* = 2.954 GHz/kOe^22^, negligible magnetocrystalline anisotropy) and a 5 × 5 × 5 nm^3^ cell size was used in the simulation.

## Additional Information

**How to cite this article**: Ding, J. *et al.* Spin Vortex Resonance in Non-planar Ferromagnetic Dots. *Sci. Rep.* **6**, 25196; doi: 10.1038/srep25196 (2016).
